# Differences in Antioxidants, Polyphenols, Protein Digestibility and Nutritional Profile between *Ganoderma lingzhi* from Industrial Crops in Asia and *Ganoderma lucidum* from Cultivation and Iberian Origin

**DOI:** 10.3390/foods10081750

**Published:** 2021-07-29

**Authors:** Raúl Fraile-Fabero, María V. Ozcariz-Fermoselle, Juan A. Oria-de-Rueda-Salgueiro, Veronica Garcia-Recio, Damian Cordoba-Diaz, María del P. Jiménez-López, Tomás Girbés-Juan

**Affiliations:** 1 Departamento Nutrición y Bromatología, Facultad de Medicina, Universidad de Valladolid, Av. Ramón y Cajal, 7, 47005 Valladolid, Spain; raulfrailef@gmail.com (R.F.-F.); mariapilar.jimenez@uva.es (M.d.P.J.-L.); 2 Departamento Ciencias Agroforestales, ETSIIAA, Universidad de Valladolid, Av. Madrid, 57, 34004 Palencia, Spain; oria@agro.uva.es; 3 Departamento Ingeniería Agrícola y Forestal, ETSIIAA, Universidad de Valladolid, Av. Madrid, 57, 34004 Palencia, Spain; mvirginiaozcariz@gmail.com; 4 Departamento Farmacia Galénica y Tecnología Alimentaria, Facultad de Farmacia, Universidad Complutense de Madrid, Plaza Ramón y Cajal, s/n, 28040 Madrid, Spain; vgrecio@ucm.es (V.G.-R.); damianco@ucm.es (D.C.-D.)

**Keywords:** *Ganoderma lingzhi*, *Ganoderma lucidum*, phenols, protein electrophoresis, protein digestibility, nutritional analysis, fatty acids

## Abstract

Carpophores of *Ganoderma lingzhi* (GZ) from industrial crops in China were analysed and compared with carpophores of three Iberian strains of cultivated *Ganoderma lucidum* (GL) (Aveiro, Madrid, Palencia) previously genetically characterized. The genetic determination of all the fungi in the study coincided with the identification provided by the companies and entities that supplied the samples. Cultivation time ranged between 107 and 141 days. The analysis of total phenol content showed to be 56.8% higher for GL from Palencia than for *GZ*. Intraspecific variation was a maximum of 56% from GL. The content of antioxidants, both intraspecific and interspecific, was found to be strain-dependent with a maximum variation of 78.5%. The nutritional analysis shows that there are differences in dietary fiber, protein, ash and sodium content between GL and GZ. In fatty acids analysis, only trans fatty acids showed significant differences, being higher in GL. Protein profile and digestibility of *GZ* and GL-Madrid mushroom proteins were evaluated by digestion with simulated gastric fluid and were different. The two species were perfectly differentiated according to their protein profile. These results should be considered for nutritional and industrial applications.

## 1. Introduction

GL is a saprophyte fungus whose carpophores have been used in traditional medicine for more than two thousand years [1]. Basidiomata of GL contains bioactive compounds, such as phenols, triterpenes, polysaccharides, proteins, enzymes and fatty acids with well-described pharmacological activities [2]. Several manuscripts documented the antitumor activity [3], the antiviral effect [4,5], and protective properties for numerous organs such as the brain [6], cardiovascular system [7,8] and liver [9] of these fungi.

The search for new sources of antioxidants has become of primary importance in contemporary science since oxidative stress can lead to various medical conditions [10]. The excess of free radicals within the human body favors the development or enhancement of many diseases such as cancer, cardiovascular problems or diabetes [11].

It is known that different strains of GL from different geographic regions have different properties, one such property being antioxidant power [12]. Despite exhibiting significant morphological differences, *GZ* has a golden colored pore surface whereas GL’s is white. They were considered the same species as GL until 2012 [13], so a review of certain pre-2012 research might be suggested [14]. A large percentage of GL currently sold in the market actually belongs to the GZ species, especially carpophores for crops from countries such as China, India or Indonesia, the largest producers of *Ganoderma* spp. This is why we consider it interesting to know the differences between both species. The genetic identification of the strains prevents errors in the determination of the species and ensures that all subsequent analyzes are correctly assigned to each. The cultivation of GL in the laboratory allows the control of characteristics such as substrate, humidity, light and temperature, whose influence could alter the content of total phenols or fungus antioxidants. The smallest fructification times indicate faster growth and greater strain vigor, a parameter of interest in industrial crops.

To date, there are very few published works comparing the GZ species predominantly cultivated in Asia with the GL of European origin. GZ has a higher content of terpene acids such as ganoderic acid A [13] and a new triterpene with cytotoxic activity, lucidumol C [15].

To our knowledge, there are no studies comparing differences in protein profile, protein digestibility, or antioxidant capacity between GL and GZ, or showing the complete nutritional profile of GZ. The aim of this study is to characterize and compare the electrophoretic protein profile and the protein digestibility of GZ and GL carpophores. In addition, antioxidant capacity and phenolic content of GZ carpophores from industrial crops from Shenzhen (China) were compared with three different GL strains from the Iberian Peninsula (Aveiro, Palencia and Madrid). Finally, total sugars, protein, ash, crude fat, dietary fiber, sodium, energy values and fatty acid profiles (46 fatty acids) were analysed.

## 2. Materials and Methods

### 2.1. Collection of Samples and Mycelium

For the study, three Iberian strains of GL were used: GL Madrid (GLM), growing on *Quercus ilex* subs. *Ballota*, was harvested in Robledo de Chavela (Madrid, Spain); GL Palencia (GLP), growing on *Quercus faginea,* was harvested in Valdespina, (Palencia, Spain). These two strains were identified by the Mycology Department of the University of Valladolid (Palencia, Spain). The third Iberian strain, GL Aveiro (GLA), from the District of Aveiro (Portugal) and growing on unknown substratum, was purchased from Fungi Perfect (Mealhada, District of Aveiro, Portugal).

Since it was not possible to obtain a live strain of GZ for a culture in parallel, GZ mushrooms were obtained from MundoReishi Salud S.L. from crops in Shenzhen, China. These fungi were cultivated in 80% broadleaf woodland and 20% cereals substrate, according to the producers.

All fungal, strain and carpophore samples were genetically analysed using a modified protocol based on the CTAB of Zhang et al. [16]. The amplification and sequencing of the ITS region of the ribosomal DNA were performed in depth. Results were analysed with BioEdit Sequence Alignment Editor Version 7.2.5 software (Carlsbad, CA, USA) and the sequences were checked and introduced into NCBI’s BLAST application to determine the specific identity of the fungi with sequences by other authors.

### 2.2. Cultivation

#### 2.2.1. Spawn Production

Spawn production was carried out in glass jars, using a mixture containing 59.1% whole wheat biological grain, 40% distilled water, 0.1% CaCO_3_ and 0.8% CaSO_4_ [17]. The substrate was sterilized in an autoclave at 121 °C for 45 min. The jars were then inoculated with 5% (5 g of mycelium × 10^−2^ g sterile substrate) and incubated at 25 °C. When the mycelium had colonized the entire jar, a second replication was carried out from the colonized grain, following the same procedure.

#### 2.2.2. Preparation of Cultivation Substrates

Two culture substrates were used. Substrate 1 was prepared to compare the antioxidant and phenolic content of the different strains and its composition was 20% whole wheat biological grain (*Triticum aestivum*) and 80% poplar wood chip (*Populus* sp.). Twelve glass jars each with a capacity of 200 cm^3^ were filled with the corresponding substrate. Substrate 2 was prepared to produce carpophores intended for protein digestibility, nutritional and fatty acid profile analysis. Its composition was 20% whole rye biological grain (*Secale cereale*) and an 80% mixture of Pyrenean oak wood chip (*Quercus pyrenaica*), contained in 4 L polypropylene bags supplied with a breathing filter. Four bags per strain were filled. All substrates were hydrated to obtain moisture between 60% and 70% with demineralized water. 0.1% CaCO_3_ on a dry weight basis was added to each substrate [18] and the substrates were autoclaved for 45 min.

#### 2.2.3. Inoculation, Growing and Recollection

The sterilized substrates were inoculated with 5% spawn production and were placed in a climatic chamber at 25 °C without light. Once the mycelium had colonized all the material, the containers were opened and kept at 25 °C, with 85% humidity and 10 h of light per day (500–700 lux). Carpophores were harvested when the basidiomata stopped growing, with the disappearance of the white growth edge. They were recollected and dried in the oven at 60 °C with forced air and stored in zipper bags at 4 °C.

### 2.3. Preparation of Samples for Analysis

For antioxidant analysis, the carpophores were ground in a mill grinder and 1 g of product was added to a 10 mL test tube containing extraction buffer (0.1 M Tris, 10 mM EDTA, pH 7.4) and incubated at 75 °C for 8 min. They were then centrifuged at 4 °C and 3500 rpm for 45 min. The resulting supernatant was separated into 2 mL microtubes and centrifuged at 103 rpm for 5 min. 1.5 mL aliquots were taken from each supernatant and stored frozen at −20 °C.

For protein extraction, the carpophores were ground in a mill grinder and 2 g of product were added to 20 mL of extraction buffer (0.1 M Tris, 10 mM EDTA, pH 7.4) and incubated at 4 °C for 24 h. They were then centrifuged at 4 °C and 3500 rpm for 10 min. The collected supernatant was centrifuged for 15 min at 10,000 rpm. The new supernatant was subsequently concentrated approximately 30 times with Amicon^®^ Ultra 0.5 mL centrifugal filters of 10 kDa molecular weight cut-off (Sigma-Aldrich, Madrid, Spain).

### 2.4. Nutritional Analysis

300 g of GLM carpophores grown on Substrate 2, and 300 g of GZ carpophores from samples from crops in Shenzhen, China, were analysed for protein, fat, dietary fiber, total sugars, carbohydrates, sodium, ash and fatty acid profile. The fatty acid profile was determined using 46 reference standards. For the protein content, the value of total nitrogen (N) determined by Kjeldahl methodology was converted to N × 4.38 fungal protein [19].

### 2.5. Analysis of Content in Total Phenols and Antioxidant Capacity

The antioxidant capacity was determined according to the CUPRAC procedure of Apak et al. (2004) [20]. Gallic acid (Sigma-Aldrich, Madrid, Spain) was used as standard. All analyses were performed in triplicate, according to the different mushrooms that came in turn from different containers. Total phenol content was determined by the Folin-Ciocalteu methodology described by Singleton and Rossi (1965) [21] using gallic acid as a standard.

### 2.6. Protein Digestibility and Electrophoretic Profile

In vitro protein digestibility studies of *Ganoderma* extracts were performed in simulated gastric fluid (SGF) at 37 °C for 60 min according to the procedure previously described by Jimenez et al. in 2013 [22]. Blank tests without proteins and with BSA were performed to optimize experimental conditions. Thereafter, 18 µL of the crude protein extracts or the samples obtained from in vitro digestion were incubated in SGF at 100 °C for 5 min with 6 µL of 4 × Laemmli sample buffer (Bio-Rad Labs. Alcobendas, Spain). Then, 20 µL of each sample was loaded into Mini-PROTEAN TGXTM Precast Gels 4–20% (Bio-Rad Labs. Alcobendas, Spain). Precision Plus Protein ™ Unstained Standards (Bio-Rad Labs. Alcobendas, Spain) was used as standard containing a mixture of ten Strep-tagged recombinant proteins (10–250 kDa), including three reference bands (25, 50, and 75 kDa). Electrophoresis was carried out at 20 °C and 25 mA per gel, using a buffer containing 25 mM Tris–HCl (pH 8.3), 192 mM glycine, and 0.1% (*w*/*v*) SDS. The gels were then stained overnight with a solution containing 1% (*w*/*v*) Coomassie Brilliant Blue R-250 [22].

### 2.7. Analysis of Data

The analysis of variance (ANOVA) was carried out with Statgraphics Centurion 16.1 (StatPoint Technologies Inc., Warrenton, VA, USA). The differences between treatments were compared using Fisher’s Least Significant Difference (LSD) test with a confidence level of 95.0%.

## 3. Results

The lengths of the base sequences obtained for the GZ carpophores and the three mycelium strains of GL (GLM, GLP and GLA) were 593, 590, 630 and 634 nucleotides, respectively. They were compared with sequences from other studies using the NCBI BLAST tool. The genetic determination of all the fungi in the study coincided with the identification provided by the companies and entities that supplied the samples.

### 3.1. Cultivation

**Figure 1:**
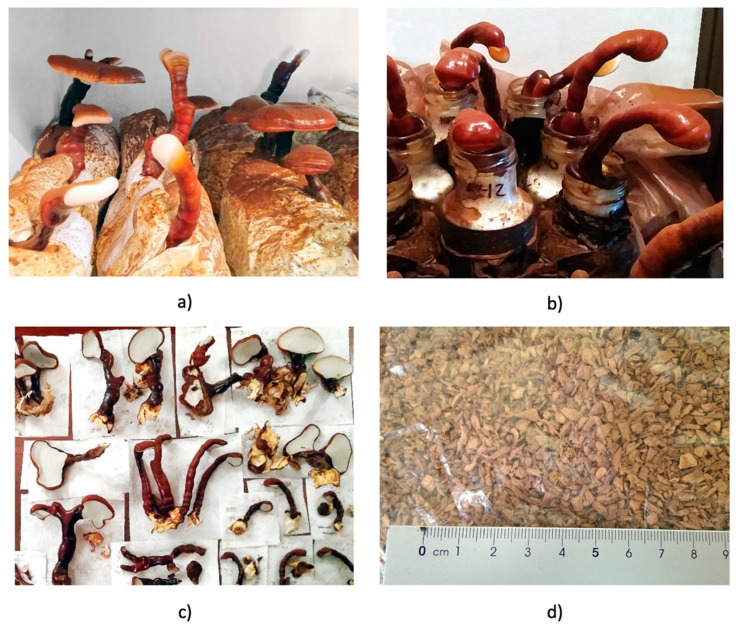
Photograph of *Ganoderma lucidum* (GL) and *Ganoderma lingzhi* (GZ) crops. (**a**) Colonized bags and fruit body production of GL; (**b**) Jars with fruiting bodies of different strains of GL; (**c**) GL mushrooms harvested and dried; (**d**) Chopped GZ carpophores.

The results of the cultivation of the different crops (Figure 1) are outlined in Table 1.

In crop 1, only the carpophores from the first flush were collected, in order to discard the potential differences in composition between different harvests. Crop 2 had a second carpophores production 59.3 ± 29 days after the first harvest and a third residual production 58 days after the second harvest. The antioxidants and phenols were only compared with the first flush. All three strains of crop 1 (GLA, GLM and GLP) had very similar results in terms of colonization and flush periods, without significant differences. However, only GLM was chosen for the second culture phase (cultivation 2) because in previous work [23] more homogeneous and faster growth has been observed (lower standard deviation, strain GL1).

### 3.2. Nutritional Analysis

The nutritional analysis showed significant differences in dietary fiber, protein, ash and sodium content between the two species (GZ and GL). GZ showed a fiber content 7.46 g per 100 g higher than GL and a sodium content 0.0024 g × 10^−2^ g higher, while GL showed a higher protein and ash content, 4.24 g × 10^−2^ g and 1.10 g × 10^−2^ g, respectively. No other significant differences were observed in the rest of the variables analysed (Table 2).

### 3.3. Fatty Acids Analysis

Among the 46 fatty acids analysed, 26 were detected in GL and 15 in GZ, all of them corresponding to those detected in GL. Twenty of the fatty acids analysed were not found in either of the two species (< 0.05%). The composition of the different components of the fatty acids found in GZ and GL were very similar, without significant differences in monounsaturated (37.5% and 28.7%), polyunsaturated (43.8% and 49.9%) and saturated (18.6% and 20.8%) fatty acids. Only trans fatty acids showed significant differences, being higher in GL. In GZ, the mixture of linoleic (43.7%), oleic (34.2%) and palmitic (13.7%) fatty acids represents 91.6% of the total. The most abundant fatty acids are the same in GL: linoleic (48.83%), oleic (25.7%) and palmitic (15.0%), amounting to 89.6% of the total. GZ contains the highest percentage of arachidic, margaric, behenic, margaroleic, lignoceric and cis-vaccenic acid, whereas GL contains the highest percentage in α-linolenic, myristic, stearic, capric, erucic, nervonic, elaidic, octadecatrienoic, octadecadienoic and eicosatrienoic acid (Table 3).

### 3.4. Analysis of Content in Total Phenols and Antioxidant Capacity

**Figure 2:**
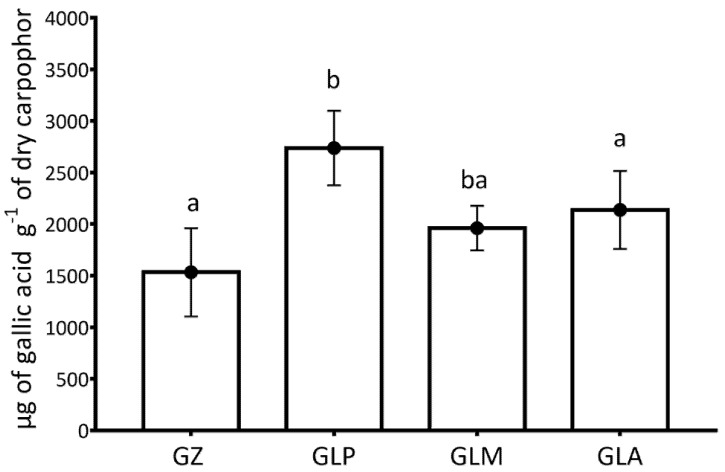
Antioxidant capacity measured in the different carpophores of GL and GZ of the CUPRAC study. Different letters indicate significant differences between taxa (*p* < 0.05). GZ: GZ from Shenzhen, China. GLA: GL from Aveiro, Portugal. GLM: GL from Madrid, Spain. GLP: GL from Palencia, Spain.

**Figure 3:**
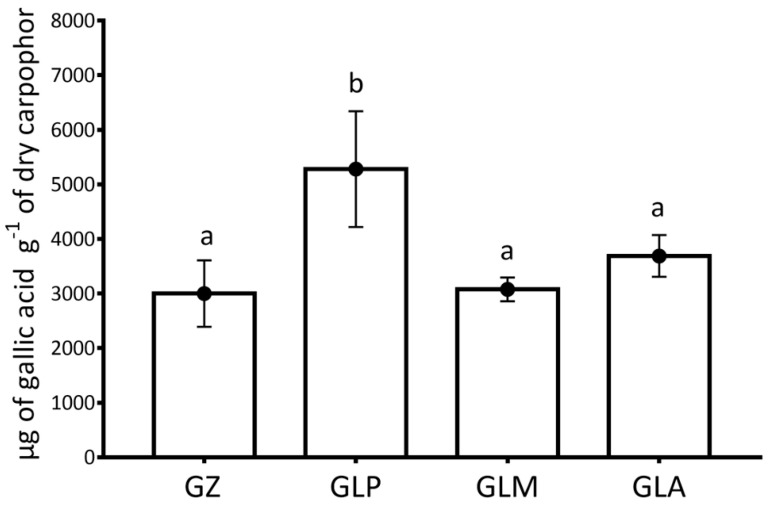
Total phenol content measured in the different carpophores of GL and GZ of the study using the Folin-Ciocalteu method. Different letters indicate significant differences (*p* < 0.05). GZ: GZ from Shenzhen, China. GLA: GL from Aveiro, Portugal. GLM: GL from Madrid, Spain. GLP: GL from Palencia, Spain.

Regression analysis from CUPRAC and Folin-Ciocalteu assay was, respectively: y = 0.0844 X + 0.0152 (*R*^2^ = 0.9985); y = 0.0381 X + 0.0066 (*R*^2^ = 0.9996). The antioxidant and polyphenol contents of GZ and GL are given in Figure 2 and Figure 3, respectively. Carpophores obtained by growing the Iberian strains of GL had similar or higher amounts of antioxidants and polyphenols than the industrial GZ carpophores from China. The analysis of total phenol content proved to be 56.8% higher for GLP than for GZ. The maximum values were obtained in the CUPRAC assay for GLP strains (78.5% higher than the mean GZ results). Regarding the antioxidant capacity of the three Iberian strains evaluated by the CUPRAC assay, the only significant differences were observed between GLP and GLM, with GLP having 39.6% more antioxidant power than GLM. Regarding phenolic content, it was higher for GLP than for GLM and GLA, differing by 71.6% and 43.1%, respectively. The last two strains showed no differences.

A positive correlation between the polyphenol content and antioxidant capacity was found for both for GL (eqCUPRAC = 789.016 + 0.368916 * eqFolin-Ciocalteu; *R*^2^ = 86.51%) and GZ (eqCUPRAC = 313.633 + 61.596 * eqFolin-Ciocalteu; *R*^2^ = 76.37%).

### 3.5. Electrophoretic Protein Profile and Digestibility

**Figure 4:**
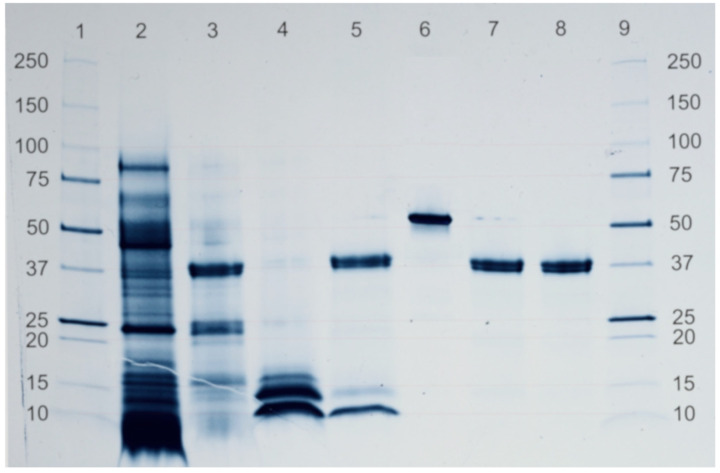
Electrophoretic profile and protein digestibility of GL and *GZ*. 1 and 9: Protein patterns. 2: GL. 3: GL digested. 4: *GZ*. 5: *GZ*. digested. 6: Bovine serum albumin. 7: Bovine serum albumin digested. 8: simulated gastric fluid.

The result of the electrophoresis and protein digestibility can be seen in Figure 4.

Molecular characterization was carried out by SDS-PAGE. As shown in Figure 4, GL has several proteins with Mr below 100 kDa, whereas *GZ* shows three bands corresponding to proteins with very similar Mr between 10–15 kDa. SGF was used to digest proteins and it was also run as a control for electrophoresis. For BSA, the digestibility process produces the total degradation of the protein, appearing only as a band of high intensity with apparent Mr of 37 kDa, corresponding to SGF. When GL is subjected to the digestibility process, most of the bands disappear; remaining are only the band corresponding to SGF and two bands with less intensity than for the undigested protein with an apparent Mr of 25 kDa and 15 kDa. The same occurs for GZ digested, mitigating the bands with higher intensity and another appearing corresponding to SGF.

## 4. Discussion

Each mushroom has unique genetic characteristics that originate a composition and particular properties. Despite belonging to the same species, each mushroom has different characteristics depending on where it is collected. The composition of each fungus is determined by the composition of the substrate, so depending on the substrate with which it works, the composition of its bioactive molecules changes. For this reason, it is important to define the identity of mushrooms to determine their nutritional and industrial value.

Although the culture in jar contained five times less substrate volume than the culture in bag, the culture in the bag colonized and fructified in much less time. Most likely, this is because the filter bags used allowed for better gas exchange and therefore better fungal development than the glass jars.

Apparently, GZ electrophoresis and protein digestion were analysed for the first time. Results show that GZ electrophoretic profile is very different from GL profile. They differ mainly in the number of proteins but also in their diversity. This technique could serve to differentiate these two species of great economic value and widely confused in the scientific literature [13].

If the GL electrophoresis is compared with results obtained by Jeurink et al., in 2008 [24] it is observed that mycelium and carpophore have a very similar protein profile. However, the 2D electrophoresis of Kumakura et al. (2018) [25] obtained with GL mushroom proteins looks very different, being more similar to GZ electrophoresis. This could be due to confusion about the taxa used as a starting point to prepare the extracts. This confusion is very frequent, especially before 2012, when they were genetically separated [26].

GL and GZ contain some proteins that are totally or partially resistant to SGF. It is accepted that pepsin-resistant proteins may have allergenic effects and some have immunomodulatory properties [27,28]. It would be interesting to know if other GL proteins resistant with 23, 16 and 15 kDa have immunomodulatory properties since they seem especially abundant.

GZ digestion has a very abundant 12 kDa resistant protein. This could be the immunomodulatory protein Ling Zhi-8 found in GL and with a molecular weight of 12,480 Da [29], but it would be necessary to sequence it to confirm this. This protein is of special interest due to its high biological activity that is currently still being investigated [30].

The GL electrophoresis of our study barely shows a 12.5kDa protein, where the Ling Zhi-8 protein would be located. It might be that Tanaka et al. (1989) [29] had worked with GZ instead of GL. In a new operation, we would have to sequence the protein to be able to obtain a conclusive result. Additionally, GZ could have a truncated version of the GL protein.

For the measurement of polyphenols and antioxidants, the wood of *Populus* (substrate 1) was chosen considering the possibility that GZ could have been cultivated in a similar substrate, since the species of *Populus* spp. are widely distributed, easy to grow and very productive. In a second phase of the study, given the impossibility of knowing the exact species used for GZ cultivation, it was chosen to select *Quercus* wood, since in the Iberian Peninsula GL appears in abundance in trees of *Quercus* and, therefore, it can be estimated that nutritional analyses, protein digestibility and fatty acid profile will better represent a wild mushroom.

The results of this study show that there are significant differences between antioxidant and phenol content of GZ and GL, and such differences are also found among the different strains of GL. Although the three strains of GL have been harvested in the Iberian Peninsula, they have been collected in different ecosystems from Spain and Portugal, where rains, soil and vegetation, among other features, are different. This, together with the geographical isolation of the strains, may have conditioned the appearance of ecotypes that explain the difference of the antioxidants between them. More studies are needed that corroborate this information.

Differences in antioxidants and phenolic content between GL and GZ are as much as 78.5% and 76%, respectively. Since the composition of the growing substrate of the fungus influences the concentration of GL antioxidants [31], we cannot discern if the differences between GL and GZ are due to the species or the substrate, since we do not know the medium of GZ culture. Mushroom drying may influence the antioxidant value of the product [32]. Therefore, since the GZ used was dehydrated and its drying process is unknown, no definitive conclusions can be drawn. Comparing the three Iberian GL strains, only for activity, the only significant difference was between GLP and GLM, with activity being 39.6% higher in the former. Stojkovic et al. 2014 compared total phenolic content in strains of GL from Serbia and China, obtaining a result for 0.37% ± 0.00 for the Serbian strains and 3.30% ± 0.03 for the Chinese strains [33]. Due to substantial differences in antioxidants content among different strains of our study and noting that a higher concentration of antioxidants can mean greater biological activity in an organism, as GL is cultivated and consumed as a medicinal food globally, the results found in this study could be of significant industrial interest.

A previous study reported differences in phenol content among eight edible species of high interest [34]. Classified in order of decreasing phenolic content measured in gallic acid equivalents, these were: *Boletus edulis* (a), *Agaricus bisporus* (b), *Cantharellus cibarius* (c), *Calocybe gambosa* (c), *Craterellus cornucopioides* (cd), *Pleurotus ostreatus* (d), *Lactarius deliciosus* (d), *Hygrophorus marzuolus* (e). The analysis put GLP at the same level as *Boletus edulis* in terms of phenolic content, while GZ has a phenolic content lower than in *Agaricus bisporus*. Other authors have reported that the total phenol content detected in GL carpophores is higher than in *Agrocybe aegerita* and *Hericium erinaceus* [35].

Several factors could influence the total phenolic content and antioxidant power, such as cultivation and dehydration procedure. For instance, if the substrate is rich in selenium, the phenolic content and the antioxidant power both increase [35]. Lyophilization is the process that best preserves these nutritional properties [32]. In the present study, GL was dried using the most aggressive method (hot air). Therefore, it is possible that the antioxidant power of the Iberian GLP carpophores would be affected. Finally, the positive correlation between the content of polyphenols and their antioxidant capacity, found for both GL and GZ in this study, has also been found by other authors and in other fungi species [36].

Meanwhile, the nutritional analysis shows that there are differences in dietary fiber, protein, ash and sodium content between GL and GZ. To date, there are no available data on the nutritional value of GZ to compare the differences found between GZ and GL. Data on the nutritional content of GL in the literature are quite diverse (Table 4).

The differences found in the dietary fiber and protein content between the two species deserves more research. In fact, cell walls of fungi are composed of chitin and cellulose and are an important source of dietary fiber. This component also includes polysaccharides including beta-glucans [(1 ⟶ 3), (1 ⟶ 6)], which have high antioxidant, antitumor [37] and immunological activity [1]. It is also documented that polysaccharides with molecular weight above 300 kDa are good prebiotics that stimulate the growth of beneficial intestinal flora [38]. Consequently, a greater amount of fiber in GL and GZ probably means a higher content of glucans, and therefore greater activity in organisms.

The comparison of nutritional values between GZ and GL indicates that GL has a 56.6% higher protein content than GZ. The total protein content of GL reported in the present work is the same as that previously reported for GL strains from Serbia (11.34% ± 1.21) and higher than the content reported from China (9.93% ± 0.26) [12]. González-Matute et al. (2011) found a protein content of 7.92% ± 0.54, similar to that obtained for GZ in this study, whereas Stamets (2005) found a higher value, 15.05% [39]. Ogbe and Obeka (2013) found a crude protein content of 17.27 ± 0.35 in wild mushrooms in Nigeria [40].

The differences in sodium (Na) between GL and GZ are probably due to the difference in sodium content of the culture substrates. It is possible that the addition of sodium to the cultivation media of the fungus resulted in its accumulation in the carpophores, as reported for selenium in the substrate for GL [35].

Linoleic acid, oleic acid and palmitic acid are the three most abundant fatty acids in GL, as Lv et al. (2012) evidenced [41]. Linoleic: oleic acid ratio could provide an important criterion from a chemo-taxonomic viewpoint and could be useful for the taxonomical differentiation among species of the same genus [42]. In this study, the ratio is 1.9 for GL and 1.28 for GZ. However, calculating the ratios of the nineteen GL samples from the study by Lv et al. (2012) from different provinces of China [41], we see that these range from 0.46 to 2.59, so we consider that this ratio does not have taxonomic validity.

To our knowledge, this is the first study where GZ and GL have been compared for nutritional differences. Results of this study indicate that both species have a different digestibility and protein profile. GL contains antioxidants and phenols in amounts that can vary considerably between the two species, as well as among the different strains of GL. Different strains, growing conditions and collecting areas may have an important influence on the nutritional value of *Ganoderma*. Consequently, more comparative studies between GZ and GL are needed to help select which of these species should be used as a source of bioactive substances in dietary supplements.

## 5. Conclusions

GL and GZ are species of different fungi, well characterized both morphologically and genetically, but confused in the international market. They present different content in polyphenols, antioxidants, protein content, fiber, digestibility and fatty acid profile. The protein profile and digestibility of GL and GZ also proved to be very different. More research should be done to clarify, as this composition difference affects its medicinal properties.

## Figures and Tables

**Table 1:** Summary of cultivation of different Iberian strains of *Ganoderma lucidum* (GL). GLA: GL from Aveiro, Portugal. GLM: GL from Madrid, Spain. GLP: GL from Palencia, Spain. T: wheat grain. Ch: poplar chips. C: whole rye grain. Qp: Pyrenean oak chips.

Crop	Strain	Substrate			Colonization	First Harvest	Total Time
		Formulation	Container	(cc)	(Days)	(Days)	(Days)
1	GLA	20%T80%Ch	Jar	200	28.0 ± 2.4 _a_	100.75 ± 2.2 _a_	128.7 ± 4.7 _a_
1	GLM	20%T80%Ch	Jar	200	26.7 ± 3.5 _a_	99.5 ± 4.3 _a_	126.5 ± 7.8 _a_
1	GLP	20%T80%Ch	Jar	200	35.6 ± 8.1 _a_	106.0 ± 7.0 _a_	141 ± 15 _a_
2	GLM	20%C80%Qp	Bag	4000	18.7 ± 2.3	79.7 ± 9.8	107.7± 19.6

Different letters indicate significant differences.

**Table 2:** Nutritional comparative chart (per 100 g of product) between GZ carpophores from industrial crops in Shenzhen (China) and GL carpophores obtained by cultivation of the Iberian strain GLM from Madrid (Spain), grown on Substrate 2:20% rye and 80% Pyrenean oak.

Analysis	*G. lingzhi*	*G. lucidum*
Dietary fibre (%)	76.81 ± 3.46 _a_	69.35 ± 3.12 _b_
Protein × 4.381 (%)	7.47 ± 0.22 _a_	11.70 ± 0.35 _b_
Carbohydrates (%)	9.88 ± 1.04 _a_	11.02 ± 1.16 _a_
Total sugars (%)	<1.00 *	<1.00 *
Ash (%)	1.21 ± 0.06 _a_	2.31 ± 0.12 _b_
Total Gross Fat (%)	1.44 ± 0.10 _a_	1.26 ± 0.09 _a_
Sodium (mg × 10^−2^ g)	0.0050 ± 0.0006 _a_	0.0026 ± 0.0003 _b_

Different letters indicate significant differences. * Detection limit.

**Table 3:** Summary table of the differences in fatty acids content found between GZ carpophores from industrial crops in Shenzhen (China) and GL carpophores obtained by cultivating the Iberian GLM strain from Madrid (Spain), grown in Substrate 2: 20% rye and 80% Pyrenean oak.

Fatty Acid	*G. lingzhi* ±15%	*G. lucidum* ±15%
Capric acid	<0.05 * _a_	0.09 _b_
Behenic acid	0.4 _a_	0.11 _b_
Erucic acid	<0.05 * _a_	0.10 _b_
Arachidic Acid	0.26 _a_	0.15 _b_
Eicosatrienoic acid (11c,14c,17c)	<0.05 * _a_	0.14 _b_
Margaric acid	0.29 _a_	0.19 _b_
Margaroleic acid	0.52 _a_	0.26 _b_
Octadecadienoic acid (9c, 12c)	<0.05 * _a_	0.09 _b_
Octadecanoic/Stearic Acid	1.3 _a_	2.17 _b_
Octadecatrienoic acid (9c, 12c, 15c)/a-Linolenic acid	0.1 _a_	0.46 _b_
Octadecatrienoic acid (9t, 12c, 15t)	<0.05 * _a_	0.09 _b_
Octadecenoic acid (11c)/cis-Vaccenic acid	1.8 _a_	1.24 _b_
Octadecenoic acid (9t)/Elaidic acid	<0.05 * _a_	0.12 _b_
Tetracosanoic/Lignoceric Acid	0.69 _a_	0.21 _b_
Tetracosenoic acid (15c)/Nervonic acid	<0.05 * _a_	0.14 _b_
Tetradecanoic Acid/Myristic	0.21 _a_	0.54 _b_
Trans fatty acids	0.1 _a_	0.31 _b_
Total Monounsaturated Fatty Acids	37.5 _a_	28.68 _a_
Total polyunsaturated fatty acids	43.84 _a_	49.93 _a_
Total saturated fatty acids	18.64 _a_	20.77 _a_

Different letters indicate significant differences. * Detection limit.

**Table 4:** Nutritional values for GL by different authors compared with the dry weight values found in our study.* Cultivated; ** Wild; NR: Not reported; FA: Fatty Acids. T: Total.

	Author	[33]	[33]	[40]	[39]	Present Study
Variable						
Origin		Serbia **	China *	Nigeria **	NR *	Spain *
Dietary Fibre (%)		NR	NR	59.2% ± 0.9	66.8	69.4 ± 3.1
Proteins (%)		11.3 ± 1.2	9.9 ± 0.3	17.3 ± 0.1	15.1	11.7 ± 0.4
Total Carbohydrates (%)		81.5 ± 1.1	78.2 ± 0.2	65.8 ± 0.2	71.0	11.0 ± 1.2
Ash (%)		2.8 ± 0.0	8.2 ± 0.1	8.7± 0.1	NR	2.3 ± 0.1
Total Gross Fat (%)		4.4 ± 0.0	3.7 ± 0.0	1.6 ± 0.1	3.5	1.3 ± 0.1
Saturated FA (% T FA^−1^)		15.7 ± 0.0	32.4 ± 0.2	NR	13.7	20.8 ± 3.1
Monounsaturated FA (% T FA^−1^)		49.6 ± 0.0	25.2 ± 0.1	NR	60.9	28.7 ± 4.3
Polyunsaturated FA (% T FA^−1^)		34.7 ± 0.0	42.4 ± 0.1	NR	25.4	49.9 ± 7.5
Sodium (mg × 10^−2^ g)		NR	NR	236.5 ± 0.0	NR	-
